# Light Guidance Aided by the Toroidal Dipole and the Magnetic Quadrupole in Silicon Slotted-Disk Chains

**DOI:** 10.1021/acsphotonics.2c01840

**Published:** 2023-02-09

**Authors:** Evelyn Díaz-Escobar, Ángela I. Barreda, Laura Mercadé, Amadeu Griol, Alessandro Pitanti, Alejandro Martínez

**Affiliations:** † Nanophotonics Technology Center, Universitat Politécnica de Valéncia, Camino de Vera s/n, 46022 Valencia, Spain; ‡ Institute of Solid State Physics, Friedrich Schiller University Jena, Max-Wien-Platz 1, 07743 Jena, Germany; § Institute of Applied Physics, Abbe Center of Photonics, Friedrich Schiller University Jena, Albert-Einstein-Str. 15, 07745 Jena, Germany; ∥ MIND-IN2UB, Departament d’Enginyeria Electrònica i Biomédica, Facultat de Física, Universitat de Barcelona, Martí i Franqués 1, 08028 Barcelona, Spain; ⊥ NEST Lab, CNR - Istituto di Nanoscienze and Scuola Normale Superiore, Piazza San Silvestro 12, 56217 Pisa, Italy

**Keywords:** silicon photonics, high-index nanophotonics, anapole states, Mie nanophotonics, photonic crystals

## Abstract

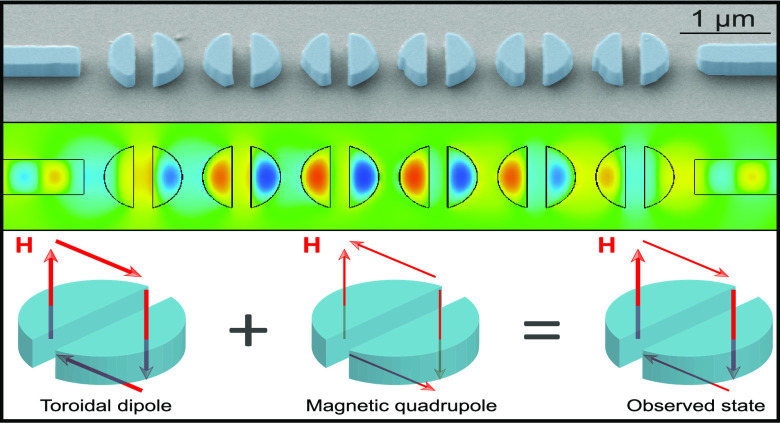

Far-field scattering of high-index nanoparticles can be hugely reduced via interference of multipolar moments giving rise to the so-called anapole states. It has been suggested that this reduced scattering can contribute to efficient transmission along periodic chains of such nanoparticles. In this work, we analyze via numerical simulation and experiments the transmission of light along chains of regular and slotted silicon disks in the frequency region over the light cone. We do not observe transmission at wavelengths corresponding to the excitation of the first electric anapole for regular disks. However, large transmission along straight and curved chains is observed for slotted disks due to the simultaneous excitation of the toroidal dipole and magnetic quadrupole modes in the disks. Photonic band calculations unveil that such large transmission can be ascribed to leaky resonances, though bound states in the continuum do not appear in the structures under analysis. Experiments at telecom wavelengths using silicon disk chains confirm the numerical results for straight and bent chains. Our results provide new insights into the role of radiationless states in light guidance along nanoparticle chains and offer new avenues to utilize Mie resonances of simple nanophotonic structures for on-chip dielectric photonics.

## Introduction

High-index dielectric nanoparticles support multipolar moments with different near-field and far-field properties, which provides a way to manipulate light at the subwavelength level.^1−3^ Under certain circumstances, multipoles displaying different near-field patterns but identical far-field scattering can interfere destructively, resulting in the so-called anapole states.^4−6^ Such scatteringless states are usually accompanied by a huge field concentration inside the nanoparticle without radiative losses,^6,7^ which enhances the light–matter interaction^8^ and results in efficient nonlinear effects.^9−11^ An easy way to build anapole states in thin high-index films—such as on silicon-on-insulator (SOI)—is by defining subwavelength-size disks using standard lithographic tools.^4^ This way, the anapole state could be employed to enhance the light–matter interaction so that the silicon disk becomes a relevant wavelength-scale building block in on-chip integrated photonics.^12,13^

The excitation of different electromagnetic multipolar moments can also lead to interesting effects when periodic chains are formed, such as the disappearance of the photonic bandgap in one-dimensional photonic crystals as a result of the interplay between the electric and magnetic dipole^14^ or the transfer of anapole states across an ensemble of nanoparticles.^15^ It has also been suggested and experimentally observed that chains of slotted disks can efficiently guide light at wavelengths of ∼10 μm using modes over the light line around the anapole state by exploiting its reduced out-of-plane scattering.^16^ However, as suggested in ref (16), this feature should be most valuable when building the chains on SOI and performing the guidance in the technologically relevant telecom wavelength regime.

In this work, we analyze numerically and experimentally the guidance of light along straight and bent chains of regular and slotted silicon disks in the 1.5 μm wavelength region. In agreement with ref (16), we find that the introduction of an air gap in the silicon disks improves the coupling and enhances the transmission efficiency. However, our results suggest that the guidance is related not to the existence of the electric anapole state but rather to the excitation of a toroidal dipole that couples adjacent disks together with a magnetic quadrupole that contributes to reducing the out-of-plane scattering. Numerical simulations of the periodic chain of slotted disks confirm the existence of a high-Q leaky resonance over the light cone. Experiments are in good agreement with numerical simulations and confirm the importance of interference between Mie modes to build on-chip photonics based on wavelength-scale disks.

## Simulation Results

**Figure 1:**
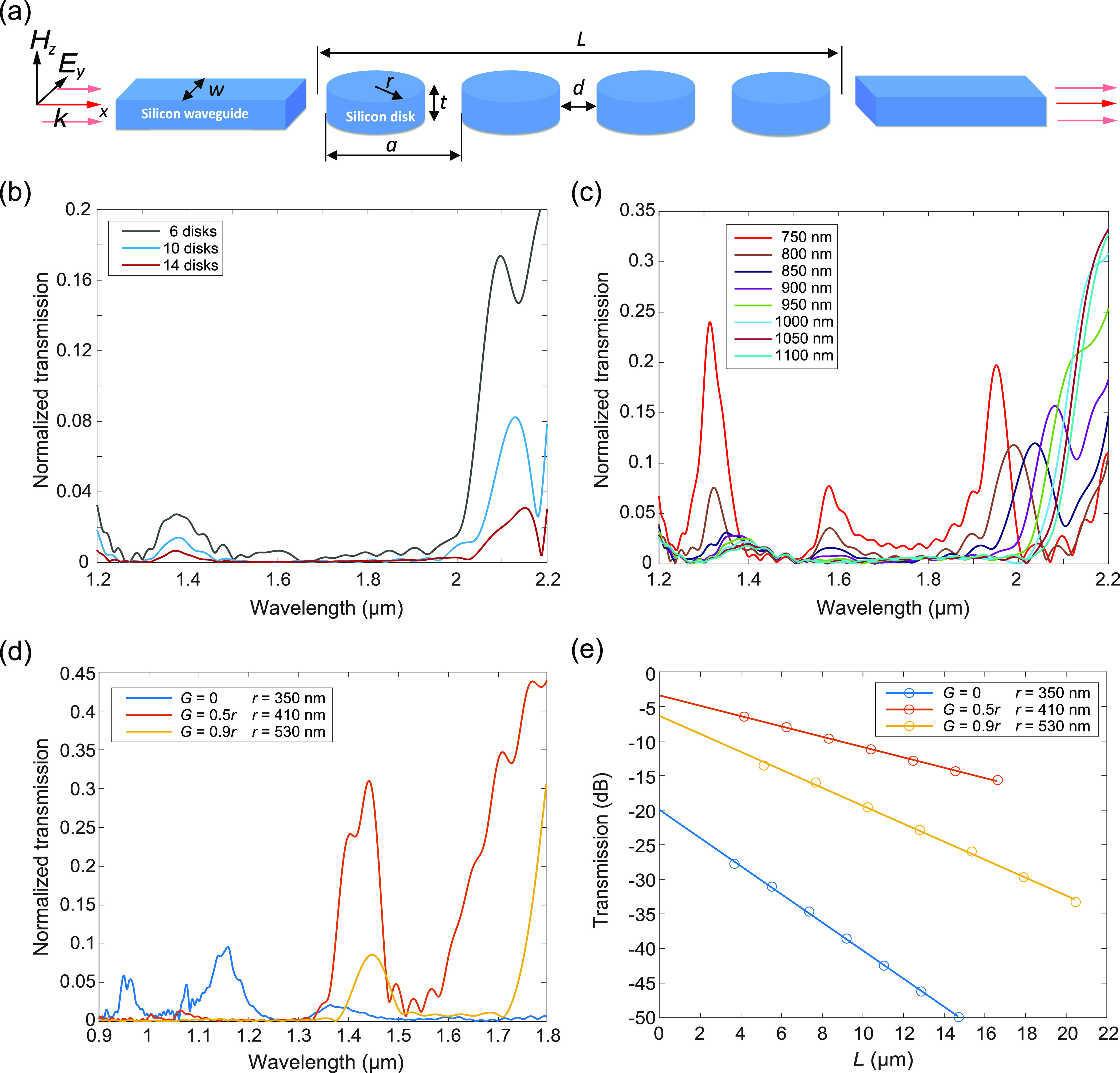
Simulations of transmission along a straight chain of silicon disks. (a) Sketch of the structure under study: an array of silicon disks (in the plot, there are *N* = 4 disks) with radius *r*, thickness *t*, and period *a* is placed between two silicon waveguides (width *w*) that act as input and output ports. (b, c) Transmission spectra normalized with respect to the response of a single waveguide (*w* = 800 nm) of (b) chains of 6, 10, and 14 disks with *r* = 350 nm, *t* = 220 nm, and *a* = 920 nm, and (c) chains of 6 disks with *r* = 350 nm and different values of the period *a*. (d) Transmission across six disks for different values of the radius *r* and the air gap *G* (see the legend) when a spacing *d* = 220 nm between disks is considered. (e) Normalized transmission as a function of the number of disks for different values of *G* (see the legend) at the wavelength of maximum transmission. The slope gives the propagation loss, and the values for *L* = 0 give the insertion loss. The simulations were performed using the commercial software CST Microwave Studio (see the Supporting Information).

We start by considering the structure sketched in Figure 1a. It consists of a chain of evenly spaced (period *a*) silicon disks with thickness *t* and radius *r*. The disks may eventually have an air gap of size *G* splitting them into two identical halves. We consider that rectangular-cross-section waveguides (width *w*) are used as input and output ports: the left-hand waveguide is used to illuminate the disk chain using the transverse electric (TE) guided mode, while the right-hand waveguide is used to collect the transmitted light. In ref (12) we verified that a single disk with *r* = 350 nm and *t* = 220 nm supports an electric anapole at wavelengths around 1.5 μm under lateral illumination. Therefore, we chose this radius to perform numerical simulations (see details in the Supporting Information) of the transmission exhibited by a disk chain. The obtained results for 6, 10, and 14 disks and *a* = 920 nm (which means that the spacing between neighboring disks is 220 nm) are depicted in Figure 1b. The transmission curves are normalized with respect to the response of a single straight waveguide. Interestingly, no appreciable transmission is observed in the region where the anapole occurs. Instead, we observe high transmission at wavelengths over 2 μm, which can be attributed to truly guided modes (or bound states^17^) below the light line (such modes can potentially appear at wavelengths >2*a*^18^) as well as in a region around 1.35 μm, which can be ascribed to higher-order Mie resonances.^12,13^ It should be noted that light guidance below the light cone along chains of high-index nanoparticles has also been reported in the literature,^14,19,20^ and the role played in the transmission by the electric and magnetic dipoles is discussed there.

We also performed calculations for different values of the period *a* to check its influence over the transmission behavior. The results, depicted in Figure 1c, show that the transmission significantly grows in the 1.5 μm wavelength region when the period *a* is reduced to values of 850 nm and below. Noticeably, the non-negligible transmission peaks for *a* values of 750 and 800 nm can be ascribed to the fact that those peaks occur in wavelength regions placed below the light cone. Still, even for a separation between disks as small as 50 nm, the transmission in the anapole region is negligible. This means that the existence of the first electric anapole state does not provide a means for efficient energy transmission along disk chains. Therefore, the absence of out-of-plane scattering is not sufficient to ensure light guidance through the chain.

This observation is consistent with the results reported in ref (16), where it is shown that transmission along a chain of perfect disks is largely attenuated around the electric anapole wavelength even when the disks are very close to each other. However, ref (16) proposed that the insertion of an air gap in the disk could contribute to reduced losses and achieve highly efficient transmission. Following this idea, we performed simulations of chains of slotted disks having air gaps of *G* = 0.5*r* and *G* = 0.9*r*, choosing the disk radius (*r* = 410 nm and *r* = 530 nm, respectively) and the interdisk spacing (*d* = 220 nm) to allocate a transmission band close to the 1.5 μm wavelength region. The results, depicted in Figure 1d, show that the insertion of the air gap gives rise to a frequency region with a relatively large transmission, especially in the case of *G* = 0.5*r*. We also computed the transmission at maxima along straight chains of different amounts of disks in the three cases shown in Figure 1d. The results, depicted in Figure 1e, show that the *G* = 0.5*r* configuration performs better in terms of insertion losses (∼3.4 dB) as well as propagation losses (∼0.75 dB/μm), in agreement with the results in ref (16).

**Figure 2:**
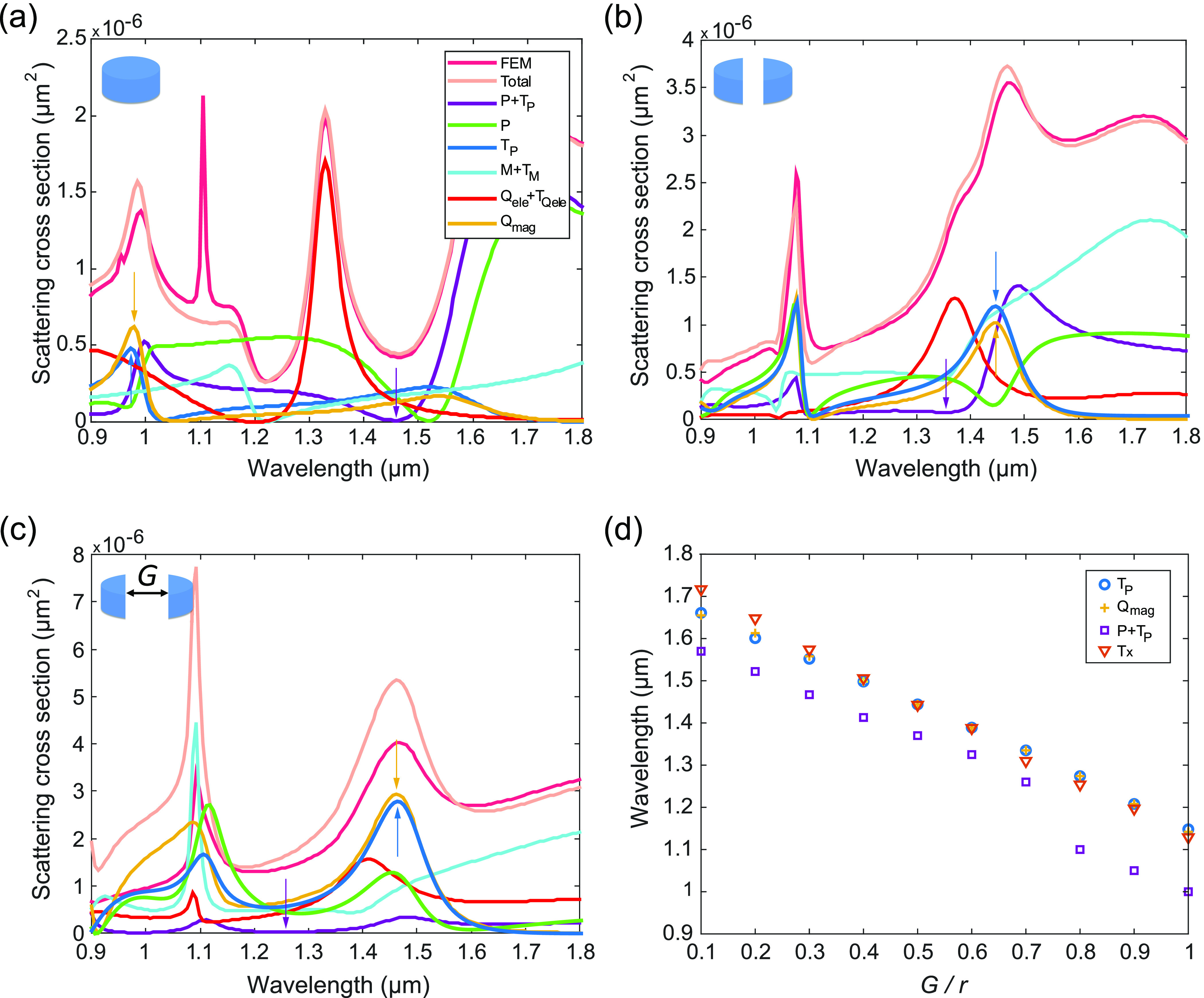
Multipole response of isolated silicon disks. (a–c) Scattering spectra of a silicon disk with (a) *r* = 350 nm and *G* = 0, (b) *r* = 410 nm and *G* = 0.5*r*, and (c) *r* = 530 nm and *G* = 0.9*r*. Spectral contributions of the electric dipole *P*, the electric toroidal dipole *T*_*P*_, and the magnetic quadrupole *Q*_mag_ under lateral illumination are presented, along with the contributions of the electric dipole plus the electric toroidal dipole (*P* + *T*_*P*_), the magnetic dipole plus the magnetic toroidal dipole (*M* + *T*_*M*_), and the electric quadrupole plus the electric toroidal quadrupole (*Q*_ele_ + *T*_*Q*_ele__). The total scattering cross-section calculated using the finite element method (FEM) are also depicted. The arrows indicate the positions of the electric anapole (*P* + *T*_*P*_ = 0), the toroidal dipole (*T*_*P*_), and the magnetic quadrupole (*Q*_mag_). (d) Calculated wavelengths of maximum chain transmission (*Tx*), electric anapole, toroidal dipole, and magnetic quadrupole as functions of the disk slot size *G*. The disk radius is *r* = 410 nm. Details about the simulations are given in the Supporting Information.

In order to establish a link between the transmission bands and the existence of anapole states in a single disk, we calculated the multipole decomposition under lateral illumination for the three different cases considered in Figure 1d. Figure 2 shows the contributions to the scattering cross section of the main multipole moments for (a) *r* = 350 nm and *G* = 0 (the case considered in ref (12)), (b) *r* = 410 nm and *G* = 0.5*r*, and (c) *r* = 530 nm and *G* = 0.9*r*. It can be seen that the wavelength regions of maximum transmission in Figure 1d also correspond to regions of large scattering (Figure 2b,c) but not to the electric anapole condition, which takes place at much shorter wavelengths. In particular, it seems that the maximum transmission along the disk chain is linked to the existence of two higher-order multipoles in an isolated disk. To confirm this assumption, we obtained the wavelength of occurrence of the different relevant states (the electric anapole, the toroidal dipole, and the magnetic quadrupole) for a single disk together with the maximum transmission wavelength along a disk chain as a function of *G*. As shown in Figure 2d, the transmission maximum perfectly overlaps with the toroidal dipole and the magnetic quadrupole, while the electric anapole is always shifted to shorter wavelengths. It is noteworthy that also in ref (16) there are a toroidal dipole mode and a magnetic quadrupole mode at the frequencies of maximum transmission.

**Figure 3:**
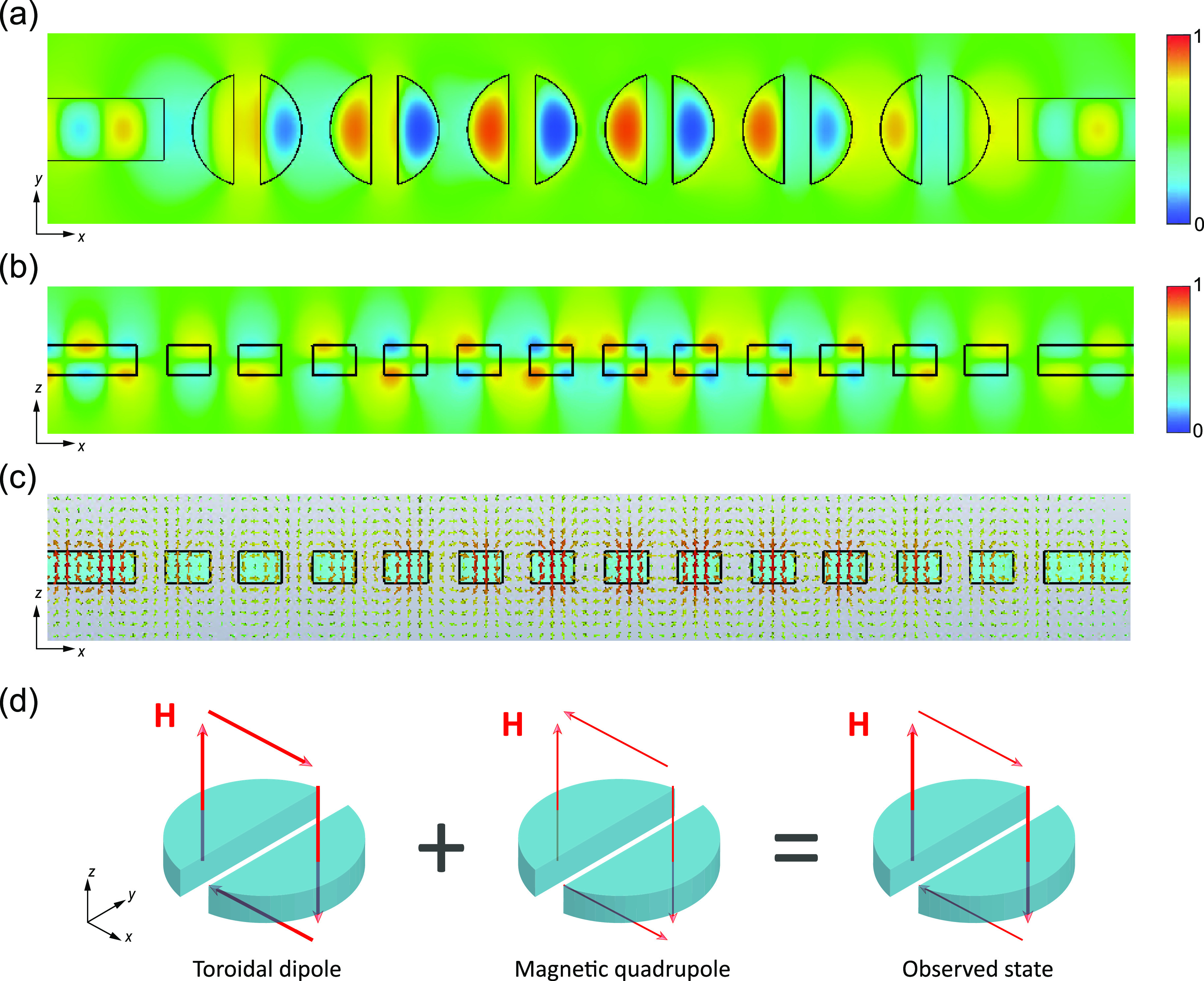
Magnetic near-fields along the slotted disk chain at maximum transmission. (a) Snapshot of *H*_*z*_ in the *xy* plane. (b) Snapshot of *H*_*x*_ in the *xz* plane. (c) Magnetic field *H* lines represented by arrows in the *xz* plane. (d) Sketch of the superposition of the magnetic field loops for the toroidal dipole and the magnetic quadrupole resulting in the observed states.

To verify the previous assumption, we performed simulations of continuous-wave signals propagating through the chains at the wavelength of maximum transmission for the case *r* = 410 nm and *G* = 0.5*r*. The results, depicted in Figure 3, show the existence of a closed loop for the magnetic field in the *xz* plane around the slotted disk. This is consistent with the excitation of the toroidal dipole,^21^ in agreement with the multipolar decomposition. In addition, the simultaneous excitation of the magnetic quadrupole results in a reduction of the magnetic field strength in the regions over and below the disk (see the arrows in Figure 3c). This should contribute to reducing the scattering along the disk axis (out-of-plane scattering), as previously shown^22,23^ and also sketched in Figure 3d.

**Figure 4:**
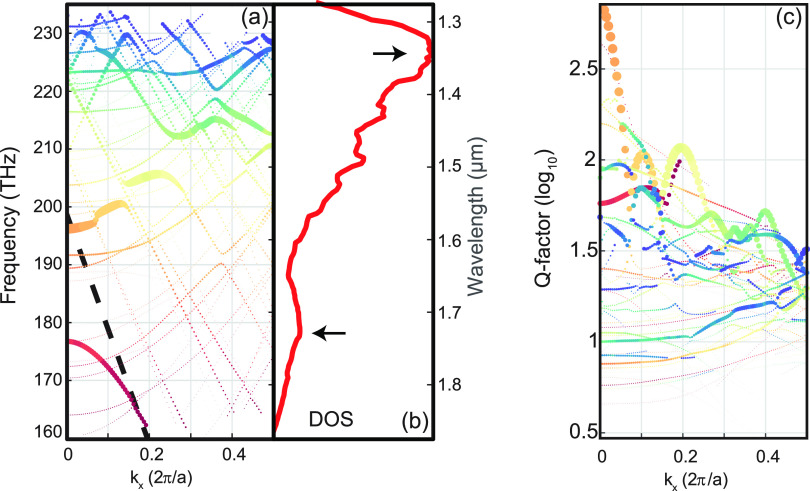
Band structure of an infinite chain of slotted disks. (a) Band structure, (b) density of States (DOS), and (c) Q-factors for an infinite chain of slotted disks with *G* = 0.5*r*. Marker size is proportional to the confinement factor of the electric field within the disk. Different modes are color-coded to aid the comparison between (a) and (c). See the Supporting Information for more details.

In ref (23), it was shown that excitation of toroidal dipoles in coupled high-index disks could eventually lead to the existence of bound states in the continuum (BICs) when two-dimensional lattices are formed. Therefore, in accordance with the results above, it makes sense to consider whether the slotted-disk chains can also support toroidal BICs and whether this could be the reason explaining the large transmission over the light line. To analyze the existence of BICs, we calculated the optical Q-factor of a periodic chain of slotted disks. Figure 4 reports the results of numerical simulations of an infinite chain of disks with *G* = 0.5*r*. To ease the visualization of the relevant modes, the size of scatter points has been chosen as directly proportional to the mode energy confinement factor within the silicon region. The band structure along the chain direction shows few regions with flat dispersion (Figure 4a), which corresponds to local maxima in the density of states (Figure 4b). These regions could correspond to peaks in the transmission, especially when combined with high Q-factors, which are reported in Figure 4c. The green flat region around λ = 1.43 μm, highlighted with a black arrow in Figure 4a, likely is mainly responsible for the strong transmission peak shown in Figure 1d, whose range between 1.32 and 1.46 μm is compatible with our simulations. Interestingly, a strong increase in Q-factor can be observed for the red-colored band around λ = 1.54 μm. This could hint toward the existence of a symmetry-protected BIC, which could be difficult to detect in transmission experiments due to its existence for nonpropagating waves at [*k*_*x*_, *k*_*y*_] = [0, 0].

## Experimental Measurements

**Figure 5:**
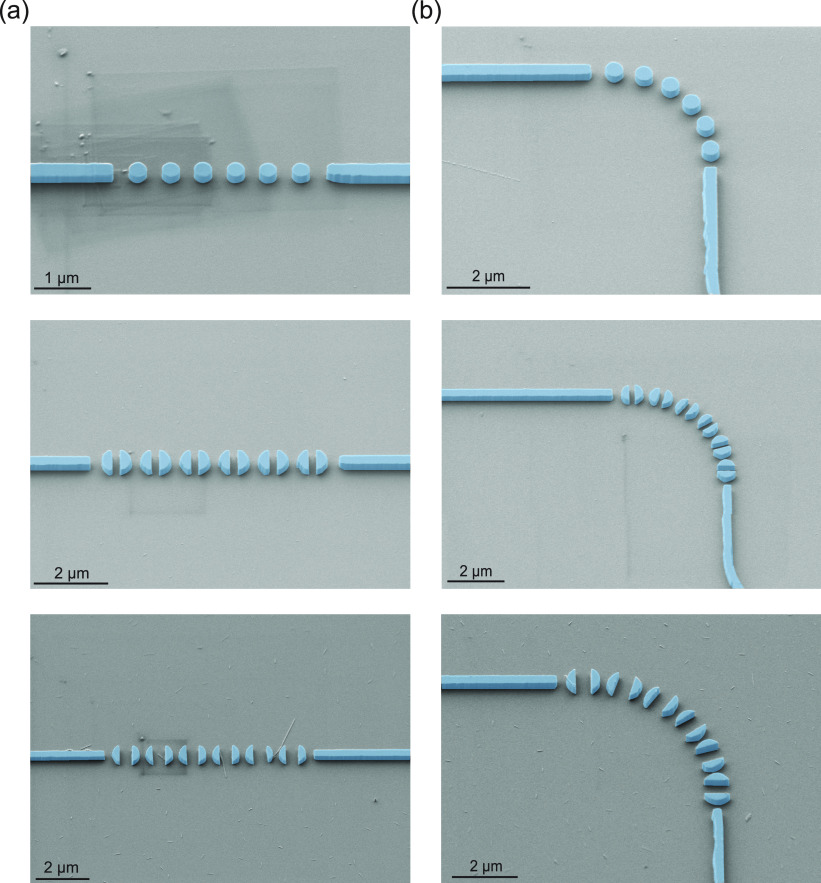
False-color SEM micrographs of several fabricated samples. Fabricated samples for six disks forming (a) straight and (b) bent chains. Rectangular-cross-section waveguides are employed as input and output ports. Circuits with *G* = 0 (top panels), *G* = 0.5*r* (middle panels), and *G* = 0.9*r* (bottom panels) were fabricated using standard silicon nanofabrication processes. The curvature radii *R* are 2060 nm for *G* = 0, 3390 nm for *G* = 0.5*r*, and 4020 nm for *G* = 0.9*r*.

**Figure 6:**
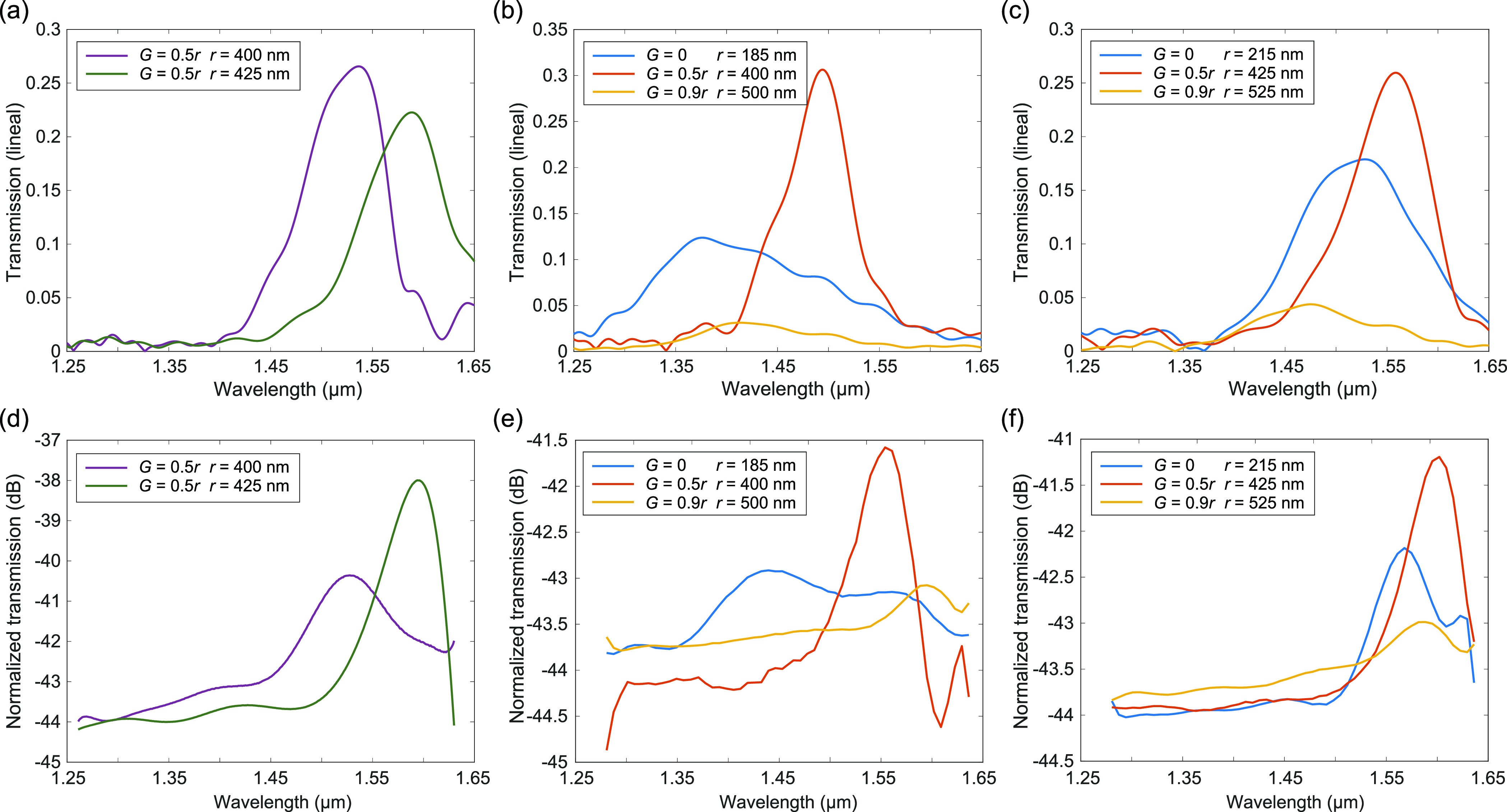
Experimental light transmission along straight and bent chains of disks. (a) Numerical and (d) experimental transmission along a straight chain of six disks with different radii *r* (nominal values shown in the panels) and air gap *G* = 0.5*r*. (b, c) Numerical and (e, f) experimental transmission along curved chains of six disks with different radii *r* and air gaps *G* (values shown in the legends). To reproduce the experimental conditions, we have considered that the silicon disk lies on a silica substrate in the simulations. Normalization in the experimental panels is performed in comparison to the input power (1 mW).

To confirm our numerical predictions, we used standard fabrication tools (see the Supporting Information) to fabricate different samples containing sets of straight and curved disk chains with waveguides as input and output ports, following the configuration sketched in Figure 1a. Figure 5 shows scanning electron microscopy (SEM) images of several fabricated circuits with different *G* values, including both straight and curved (curvature radius *R*) chains, highlighting in detail the waveguide ends acting as input and output ports as well as the disk chains. The waveguides were adiabatically widened up to 3 μm to reduce coupling losses from the input lensed fiber as well as to the output detection system^24^ (see details in the Supporting Information). We also performed numerical simulations including the silica substrate (Figure 6a–c) to compare with the results of the experimental measurements.

Figure 6d shows the measured normalized transmission for two straight chains with nominal disk radii of 400 and 425 nm with air gap *G* = 0.5*r* (Figure 5a, middle panel) separated by a 220 nm gap. Coupling losses (from fiber to waveguide) were about 15 dB per facet, and additional losses are due to some imperfections induced by problems in the etching of the disks (see the SEM images in Figure 5). Even though the noise level in our measurement system was about −44 dBm, we clearly observe a region with large transmission, in good agreement with the results obtained in the simulation depicted in Figure 6a for the two radii under consideration. It should be noted that the transmission region is red-shifted in comparison to the results of Figure 2, which we ascribe to the disk chain resting on a silica substrate.

We also performed experimental measurements on the bent chains with different air gaps in the disks, as shown in Figure 5b. The air gap of each disk was properly rotated to follow the curvature of the chain and keep perpendicular to the curve. No further engineering on the position of the disks was performed to improve the optical transmission. We included chains with disks having *r* ≈ 200 nm and *G* = 0 for comparison purposes. Figure 6e,f shows the measured normalized transmission for bent chains with three different air gaps: *G* = 0, *G* = 0.5*r*, and *G* = 0.9*r*, each for two different nominal radii disks (shown in the panels). Again, we observe a wavelength region with large transmission, which confirms the results of the numerical simulations presented in Figure 6b,c as well as the experimental results on sharp bends reported in ref (16). Indeed, our results also confirm that the disk chains with *G* = 0.5*r* show the best performance. Using numerical simulations, we found that the bending losses were ∼2 dB for *G* = 0.5*r* and a radius of curvature of 3390 nm (see the right-middle panel in Figure 5). This is slightly worse than the simulation results reported in ref (16), which can be explained by considering that the spacing between adjacent disks is larger in our case to ensure that the chain can be fabricated using standard silicon nanofabrication. We believe that the curvature losses could be further reduced by reducing the distance between adjacent slotted disks as well as by properly choosing the rotation of the slot in each disk. In general, however, these results show that toroidal-aided guidance along chains of subwavelength dielectric scatterers is also an interesting mechanism enabling low-loss guidance over sharp bends.

## Conclusion

We have analyzed the guidance of light along periodic chains of silicon disks in the technologically relevant telecom wavelength regime. We have found that chains of perfect disks do not transport energy at wavelengths corresponding to the electric anapole state. This observation is indeed consistent with the fact that the disk does not efficiently scatter radiation at the anapole wavelength and therefore cannot excite the next adjacent disk. When the disk is split into two halves by an air gap of width *G*, energy transport along chains can be relatively large, even for bent chains. Multipolar decomposition as well as near-field patterns obtained by simulations suggest that the toroidal dipole is responsible for the guidance, whereas the excitation of the magnetic quadrupole contributes to the reduction of out-of-plane scattering.^22,23^ Calculations of the Q-factor of an infinite chain show that the large transmission can also be interpreted as a leaky resonance in the continuum, but there are no signatures of a BIC. In this sense, we could envisage that further engineering of the disk could lead to the emergence of accidental BICs in one-dimensional periodic systems. Such states should be experimentally observable as high-Q resonances under lateral waveguide excitation without recurring to complex methods for vertical excitation.^25^ Experimental measurements on samples fabricated using standard silicon nanofabrication tools confirm the simulation results. Our results highlight the potential of interference between different multiple moments using very simple and compact elements as wavelength-sized disk resonators to build complex functionalities in integrated photonics.

## Supporting Information Available

Details of numerical simulations, the fabrication process, and transmission measurements (PDF)
