# Supplementary material for: Light Guidance Aided by the Toroidal Dipole and the Magnetic Quadrupole in Silicon Slotted-Disk Chains
Source: ACS Photonics. 2023 Feb 9;10(3):707–14. doi: 10.1021/acsphotonics.2c01840 (PMC10021020; doi:10.1021/acsphotonics.2c01840)
Supplement: Supplementary file 1 — ph2c01840_si_001.pdf [file ph2c01840_si_001.pdf]

Light guidance aided by the toroidal dipole and the magnetic quadrupole in silicon slotted-disk chains: Supplementary Information

Evelyn Díaz-Escobar,[†] Ángela I. Barreda,[‡,¶] Laura Mercadé,[†,§] Alessandro Pitanti,[‖] and Alejandro Martínez[*,†]

†*Nanophotonics Technology Center, Universitat Politècnica de València, Camino de Vera s/n, 46022, Valencia, Spain*

‡*Friedrich Schiller University Jena, Institute of Solid State Physics, Max-Wien-Platz 1, 07743 Jena, Germany*

¶*Institute of Applied Physics, Abbe Center of Photonics, Friedrich Schiller University Jena, Albert-Einstein-Str. 15, 07745 Jena, Germany*

§*MIND-IN2UB, Departament d'Enginyeria Electrònica i Biomèdica, Facultat de Física, Universitat de Barcelona, Martí i Franquès 1, Barcelona 08028, Spain*

‖*NEST Lab., CNR - Istituto di Nanoscienze and Scuola Normale Superiore, piazza San Silvestro 12, 56217 Pisa, Italy*

E-mail: amartinez@ntc.upv.es

Numerical Simulations

Numerical simulations in Figs. 1, 3 and 6 have been performed using the commercial 3-D full-wave solver CST Microwave Studio, which implements a finite integration technique (FIT). The FIT approach involves resolving Maxwell's equations in integral form, in contrast to most numerical methods that solve them in a differential form. A refractive index $n = 3.45$ has been considered to model the silicon structures. A hexahedral mesh with 10 cells per wavelength has been used. Open boundary conditions (perfectly matched layer) have been selected for external facets. The system has been considered to be surrounded by air in Fig. 1, and 3 and resting on silica ($n = 1.45$) in Fig. 6. Field monitors have been used to observe the fields through and around the disk.

Multipolar decomposition calculations in Fig. 2 have been performed by means of a finite-element method implemented in the commercial software COMSOL Multiphysics. In particular, we use the Radio Frequency Module that allows us to formulate and solve the differential form of Maxwell's equations (in the frequency domain) together with boundary conditions. The disk is placed at the center of a spherical homogeneous region filled with air, whose radius is $\lambda/2$. A perfectly matched layer domain, with thickness $\lambda/4$, is positioned outside of the embedding medium domain and acts as an absorber for the scattered field. The mesh is chosen sufficiently fine as to allow numerical convergence of the results. In particular, the element size of the mesh of the embedding medium is smaller than $\lambda/5$ and that of the particles is smaller than $\lambda/[3\Re(n)]$, being n the silicon refractive index. The different multipolar contributions were obtained by integrating the displacement current induced inside the nanoparticle. The energy inside the disk corresponds to the volume average of the energy density time average at the disk volume.

The band structure simulations of Fig. 4 have been implemented through a commercial finite-element-method simulator (COMSOL). A single unit cell along the x direction has been simulated using Floquet-Bloch periodic boundary conditions to span the region of reciprocal space shown in Fig. 3. Along the other two directions, perfectly matched layers

have been included which allows us to include radiative losses in the simulation. The complex eigenvalues returned by the software have been used to evaluate the mode frequency (real part of eigenfrequency) and Q-factors (real part of eigenfrequency divided by two times the imaginary part).

Concerning the visual representation of Fig. 4, the scatter point in panel (a) has been colored, at each k_x, according to their frequency value using a spectral colorscale (from red to violet). The points belonging to the same mode in panel (a) and (c) at a certain k_x has been then colored in the same way, to give an easy identification of the Q-values of different bands. To highlight a set of modes, we also plotted the marker size in pixel, as 10xΓ^2, where Γ is the electric field confinement factor in the silicon region. This gives us a very small size for strongly radiative modes whilst clearly identifying the most relevant ones for our waveguide system.

Fabrication

The disk and waveguide structures were fabricated on standard silicon-on-insulator (SOI) chips with a top silicon layer thickness of 220 nm (resistivity $\rho \sim 1 - 10\Omega\text{cm}^{-1}$, with a lightly p-doping of 1 x 10^{15}cm^{-3}) and a buried oxide layer thickness of 3 μm. The fabrication is based on an electron beam direct writing process performed on a coated 100 nm hydrogen silsesquioxane (HSQ) resist film. The mentioned electron beam exposure, performed with a Raith150 tool, was optimized in order to reach the required dimensions employing an acceleration voltage of 30 keV and an aperture size of 30 μm. After developing the HSQ resist using tetramethylammonium hydroxide as the developer, the resist patterns were transferred into the SOI samples employing an optimized inductively-coupled plasma reactive ion etching process with fluoride gases.

Two samples were fabricated: one with straight chains and the other with bent chains. The first one had two working circuits, and the second had six, each composed of an input

waveguide acting as an excitation port, six disks, and an output waveguide. For straight chains, disks of nominal radius 400 and 425 nm with $G = 0.5r$ were fabricated. For bent chains, disks of nominal radius 185, 215 nm with $G = 0$; 400, 425 nm with $G = 0.5r$; and 500, 525 nm with $G = 0.9r$ were fabricated. Details are given in Table S1.

Table S1: Dimensions of measured samples.

	Straight chains		Bent chains					
	$G = 0.5r$		$G = 0$		$G = 0.5r$		$G = 0.9r$	
Estimated disk radius r	400 nm	425 nm	185 nm	215 nm	400 nm	425 nm	500 nm	525 nm

Transmission measurements

In the reported measurements, light was generated with a SANTEC TSL-210F laser, which is tunable in the wavelength range between 1260 nm and 1630 nm. A fiber polarizer was used to ensure that only the TE-like mode was excited in the input waveguide, which was carried out via a lensed fiber. The lensed fiber and the sample were placed in positioning platforms. An objective, polarization filter, and splitter are located at the output of the chip. A polarization filter was used to select the right mode in the waveguide. To inject the TE-like mode, the filter was placed vertically, and the fiber polarizer was rotated until the power detected by the camera was zero, resulting in an estimated ratio of TE-like mode of better than 100:1. The splitter was employed to divide the light beam into two orthogonal directions, one of which was directed to a near-infrared camera to locate the output light spot and the other to a power meter (Newport 1930C). An averaging of seven intensity measurements per wavelength was carried out to reduce the ripple in the measured transmission, and then a smoothing of the data was performed by “polyfit” and “polival” Matlab functions, as done in Ref.[1]

References

(1) Espinosa-Soria, A.; Pinilla-Cienfuegos, E.; Díaz-Fernández, F. J.; Griol, A.; Martí, J.; Martínez, A. Coherent Control of a Plasmonic Nanoantenna Integrated on a Silicon Chip. *ACS Photonics* **2018**, *5*, 2712–2717.
